# *Sparassis crispa* and a Bioactive Compound Therein, Ergosterol, Were Effective in Preventing Acetylcholinesterase Inhibition In Vitro and In Vivo

**DOI:** 10.1002/fsn3.71653

**Published:** 2026-04-06

**Authors:** Chan Kyu Park, Gyumin Kang, Soo Jung Choi, Yoon Seo Lee, Eui‐Cheol Shin, Hyo In Kim, Jinbong Park, Nam Su Oh, Hyung Taek Cho, Tae Gyun Kim, Jeong Hoon Pan, Dong‐Hoon Shin, Young‐Jun Kim, Jae Kyeom Kim

**Affiliations:** ^1^ Department of Food and Biotechnology Korea University Sejong Republic of Korea; ^2^ Department of Food Regulatory Science Korea University Sejong Republic of Korea; ^3^ Food Safety Evaluation Department, Novel Food Division National Institute of Food and Drug Safety Evaluation Cheongju Republic of Korea; ^4^ Department of GreenBio Science Gyeongsang National University Jinju Republic of Korea; ^5^ Department of Pharmacology, College of Korean Medicine KyungHee University Seoul Republic of Korea; ^6^ Department of Surgery, Beth Israel Deaconess Medical Center Harvard Medical School Boston Massachusetts USA; ^7^ The Bioinformatix Gwangmyeong Republic of Korea; ^8^ Department of Food and Nutrition Chosun University Gwangju Republic of Korea; ^9^ Department of Health Behavior and Nutrition Sciences University of Delaware Newark Delaware USA

**Keywords:** acetylcholinesterase inhibition, cognitive impairment, ergosterol, oxidative stress, *Sparassis crispa*

## Abstract

Alzheimer's disease (AD) is characterized by multifactorial pathological processes, including cholinergic dysfunction and oxidative stress, highlighting the need for safer, multi‐target interventions beyond current synthetic acetylcholinesterase (AChE) inhibitors. In the present study, we screened ethanolic extracts from various edible and medicinal plants to identify natural sources with cholinesterase‐modulating activity and found that *Sparassis crispa* ( *S. crispa* ) extract exhibited robust AChE inhibitory activity. The extract also demonstrated significant antioxidant capacity and protected rat pheochromocytoma cells against oxidative stress–induced cytotoxicity. Chemical characterization using gas chromatography–mass spectrometry identified ergosterol as a major bioactive constituent of *S. crispa* extract. Ergosterol directly inhibited AChE activity in vitro and was subsequently evaluated in vivo using a trimethyltin chloride (TMT)–induced mouse model of cognitive impairment. Dietary supplementation with *S. crispa* extract significantly improved spatial working memory and attenuated TMT‐induced elevation of brain AChE activity. Notably, ergosterol supplementation produced dose‐dependent improvements in both spatial working memory and aversive learning, accompanied by restoration of cholinergic function and reduction of lipid peroxidation in brain tissues. No signs of hepatic toxicity were observed following ergosterol administration. Collectively, these findings demonstrate that *S. crispa* extract exerts cognitive benefits through combined modulation of cholinergic dysfunction and oxidative stress, and identify ergosterol as a key bioactive contributor to these effects. This study provides mechanistic insight into the neuroprotective potential of *S. crispa* and supports its development, together with ergosterol, as functional food–derived candidates for the prevention or mitigation of cognitive decline.

## Graphical abstract

*Sparassis crispa* extract exhibited acetylcholinesterase inhibitory and antioxidant activities, and ergosterol was identified as a major bioactive sterol. In a TMT‐induced mouse model, both the extract and ergosterol improved spatial working memory and learning retention while reducing brain AChE activity and lipid peroxidation. These findings suggest that *S. crispa* –derived ergosterol may contribute to cognitive improvement through modulation of cholinergic activity and oxidative stress.

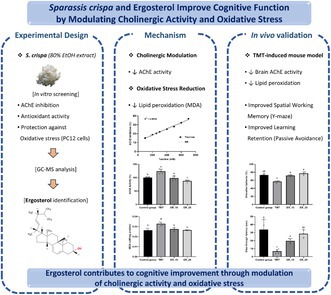

## 1 Introduction

Alzheimer's disease (AD), the most common form of dementia, imposes an enormous medical and socioeconomic burden worldwide, especially as the global elderly population rapidly increases. The pathogenesis of AD is complex and multifactorial—involving accumulation of amyloid‐β (Aβ) plaques, hyperphosphorylation of tau protein, synaptic loss, neuroinflammation, and oxidative stress (Tönnies and Trushina 2017). Among the multiple hypotheses proposed, the cholinergic hypothesis remains a central dogma: loss of cholinergic neurons in the cerebral cortex and hippocampus leading to decreased acetylcholine (ACh) levels is believed to contribute significantly to cognitive decline. Accordingly, acetylcholinesterase (AChE) inhibitors (e.g., donepezil, rivastigmine, galantamine)—which prevent synaptic ACh degradation—have been widely used as standard symptomatic treatments (Grossberg 2003). However, these synthetic AChE inhibitors are often associated with gastrointestinal (nausea, vomiting, or diarrhea), cardiovascular (bradycardia), or other side effects; moreover, they primarily provide symptomatic relief without sufficiently halting disease progression (Ruangritchankul et al. 2021). Therefore, there is an ongoing demand for safer, natural product‐based therapeutic candidates with multi‐target mechanisms that not only modulate cholinergic transmission, but also address oxidative stress, neuroinflammation, and neurodegeneration more broadly.

Edible and medicinal mushrooms have long been used in traditional medicine and cuisine, offering a rich source of bioactive compounds with reported antioxidant, anti‐inflammatory, immunomodulatory, metabolic, and anticancer effects. Among these, *Sparassis crispa* (commonly known as the cauliflower mushroom) has garnered attention for its high β‐glucan content, potent antioxidant activity, and other pharmacological properties. Specifically, recent studies suggest that *S. crispa* may extend beyond general health benefits, and hold potential as a neuroprotective agent relevant for dementia prevention. For example, in a comparative study of three mushrooms (including *S. crispa* ), *S. crispa* exhibited substantial total phenolic content, strong free‐radical scavenging activity, and measurable AChE inhibitory activity (Lee et al. 2013). Additionally, extracts of *S. crispa* have been shown to exert neuroprotective effects in hippocampal neuronal cells under glutamate‐induced oxidative stress; ethanol‐derived fractions reduced reactive oxygen species (ROS), suppressed apoptosis, and activated signaling pathways (e.g., AKT/Nrf2, and ERK/CREB) leading to upregulation of neurotrophic factors such as BDNF (Pak and Li 2023). Moreover, a polysaccharide from *S. crispa* improved cognitive function in Alzheimer‐like mouse models, accompanied by reduced Aβ deposition, suppressed neuroinflammation, and alterations in gut microbiota—suggesting involvement of the microbiota–gut–brain axis (Pak and Li 2023). These lines of evidence suggest that *S. crispa* may not act as a single‐target AChE inhibitor comparable to synthetic drugs, but rather function as a multi‐target, disease‐modifying functional food: combining moderate cholinesterase inhibition, antioxidant, and anti‐inflammatory effects.

Although these findings indicate that *S. crispa* may exert multi‐target neuroprotection, no previous study has identified the active chemical constituents responsible for its AChE inhibitory effect, and the molecular basis linking its traditional usage to cholinergic modulation remains unresolved. This lack of mechanistic clarity has hindered the translation of *S. crispa* into standardized functional materials or therapeutic candidates. To this end, in the present work, we isolated the AChE‐inhibitory component from *S. crispa* using gas chromatography–mass spectrometry (GC–MS) and identified the active molecule as ergosterol. Following this, we have implemented two in vivo behavioral tests (i.e., Y‐maze and passive avoidance test) to examine if *S. crispa* extract and its bioactive exhibit preventive potentials in vivo.

## 2 Materials and Methods

### 2.1 Cell Culture Conditions

The rat pheochromocytoma (PC12) cell line (CRL‐1721) was obtained from the American Type Culture Collection (ATCC; Manassas, VA, USA). The cells were maintained in RPMI‐1640 medium supplemented with 10% heat‐inactivated horse serum, 5% fetal bovine serum, and 1% antibiotic–antimycotic (Gibco‐Invitrogen; Grand Island, NY, USA). All inorganic salts, including sodium bicarbonate, sodium chloride, disodium hydrogen phosphate, potassium dihydrogen phosphate, and potassium chloride, were purchased from standard commercial suppliers. PC12 cells were cultured in 100‐mm tissue culture dishes and incubated at 37°C in a humidified atmosphere containing 5% CO_2_. Cells were sub‐cultured when they reached 80%–90% confluence, and the culture medium was refreshed at least three times per week.

### 2.2 Measurement of AChE Inhibition Activity

AChE activity was measured using a microplate‐based spectrophotometric method as we described elsewhere (Ellman et al. 1961; Ingkaninan et al. 2003). As a positive reference, tacrine was used. The percentage of AChE inhibition was calculated using the following formula: Activity (%) = [1 − (sample reaction − sample control)/(enzyme reaction − enzyme control)] × 100.

### 2.3 Measurement of Cytotoxicity

Cytotoxicity of the test samples was assessed using the 3‐(4,5‐dimethylthiazol‐2‐yl)‐2,5‐diphenyltetrazolium bromide (MTT) assay, as described previously (Park et al. 2025).

### 2.4 Preparation of Plant Extracts for Screening

Ten dried plant materials were purchased from an oriental medicine distributor in Seoul, Republic of Korea. The scientific names of all samples are listed in Table 1. Because *S. crispa* is a widely distributed edible mushroom commonly used as a food ingredient, a voucher specimen was not deposited. The identity of each plant material was verified based on morphological characteristics by an expert from the Department of GreenBio Science, Gyeongsang National University. All samples were stored at 4°C prior to extraction.

**TABLE 1:** Acetylcholinesterase (AChE) inhibitory activity of ethanolic extracts from various plants.

Scientific name	Acetylcholinestearse (AChE) inhibitory activity (%)
*Scutellaria baicalensis* Georgi	6.74 ± 3.10^c^
*Morus alba L*.	13.87 ± 1.64^a^
*Tricholoma matsutake*	13.45 ± 1.22^ab^
*Perilla frutescens*	7.12 ± 2.42^c^
*Euryale ferox* Salisb.	12.74 ± 1.08^b^
*Carthamus tinctorius*	8.10 ± 0.62^c^
*Cucurbita moschata* Duchesne	9.09 ± 2.52^c^
*Phellinus linteus*	11.58 ± 0.86^b^
*Ganoderma lucidum*	13.95 ± 2.73^a^
***Sparassis crispa***	**16.01 ± 3.97** ^**a**^
Tacrine (an AChE inhibitor)	14.56 ± 1.24^a^

*Note:* AChE inhibitory activity was calculated using the formula: Activity (%) = [1 − (sample reaction − sample control)/(enzyme reaction − enzyme control)] × 100. All plant extracts were tested at 1 mg/mL, and tacrine (100 nM) was used as a positive control. Each value represents the mean ± standard deviation (*n* = 4) from independent experiments. Statistical significance was evaluated using one‐way analysis of variance, followed by Dunnett's post hoc test. Different superscript letters (a–c) indicate significant differences among groups (*p* < 0.05). Bold values indicate statistically significant differences (p < 0.05) and highlight the extract with the highest acetylcholinesterase (AChE) inhibitory activity among the 10 ethanolic plant extracts screened.

Abbreviation: AChE, acetylcholinesterase.

Approximately 50 g of each dried material was pulverized into a fine powder and extracted with 80% aqueous ethanol at a sample‐to‐solvent ratio of 1:10 (w/v). An 80% hydroethanolic solvent was selected based on its established efficacy in extracting a broad spectrum of bioactive compounds from natural materials. Hydroethanolic systems containing 70%–80% ethanol are widely employed in phytochemical investigations because their intermediate polarity facilitates recovery of both moderately polar phenolic constituents and relatively less polar compounds such as sterol derivatives (e.g., ergosterol). Compared with water or absolute ethanol alone, 80% ethanol can improve extraction efficiency by enhancing cellular penetration and solubilization of structurally diverse metabolites. In addition, ethanol is a food‐grade solvent, supporting the relevance of the extract for potential functional food applications. The mixture was sonicated for 20 min in an ultrasonic bath with continuous nitrogen gas purging, and the extraction procedure was repeated two additional times. The combined extracts were filtered through Whatman No. 2 ashless filter paper using vacuum suction with a chilled Büchner funnel. Filtrates were concentrated under reduced pressure at 39°C–40°C using a rotary evaporator (EYELA; Tokyo, Japan), and the resulting dried extracts were stored at −20°C until further use. The extraction yield of *S. crispa* was 29.9% (w/w), calculated based on the dry weight of the starting material relative to the weight of the dried extract obtained after solvent evaporation. For the screening assays, each extract was dissolved in 5% dimethyl sulfoxide to a final concentration of 1 mg/mL.

### 2.5 Total Phenolic Content (TPC) Assay

TPC was measured using the Folin–Ciocalteu method as reported previously (Singleton et al. 1999). Gallic acid standards (0–200 μg/mL) and sample solutions were prepared in 15% dimethyl sulfoxide. Each standard or sample was mixed with 2% Na_2_CO_3_ and reacted for 3 min, followed by addition of 50% Folin–Ciocalteu reagent. After a 3‐min incubation at room temperature in the dark, absorbance was measured at 760 nm using a microplate reader (SpectraMax M2, Molecular Devices; San Jose, CA, USA). TPC values were calculated from the gallic acid calibration curve and expressed as gallic acid equivalents (GAE).

### 2.6 ABTS Radical Scavenging Assay

The 2,2′‐azino‐bis (3‐ethylbenzothiazoline‐6‐sulfonic acid) diammonium salt (ABTS) radical scavenging activity was measured using a microplate‐based assay (Re et al. 1999). ABTS (2.5 mM) and AAPH (1 mM) were dissolved in phosphate buffered saline, heated at 70°C for 1 h to generate the ABTS^+^• radical, and cooled to room temperature before use. Vitamin C standards (0–100 μg/mL) and sample solutions were prepared in distilled water. For the assay, 10 μL of each standard or sample was added to a 96‐well plate, followed by 190 μL of the ABTS working solution (200 μL for blanks). After a 10‐min incubation in the dark at room temperature, absorbance was measured at 734 nm using a microplate reader (SpectraMax M2, Molecular Devices). Antioxidant capacity was calculated from the standard curve and expressed as vitamin C equivalents (VCE).

### 2.7 2,2‐Diphenyl‐1‐Picrylhydrazyl (DPPH) Radical Scavenging Assay

The DPPH radical scavenging activity was determined using a microplate‐based assay (Brand‐Williams et al. 1995). A 200 μM DPPH solution was prepared in ethanol and protected from light. Vitamin C standards (0–100 μg/mL) and sample solutions were serially diluted in ethanol. In a 96‐well plate, 20 μL of each standard or sample was mixed with 180 μL of the DPPH solution. After a 30‐min incubation in the dark at room temperature, absorbance was measured at 517 nm using a microplate reader (SpectraMax M2, Molecular Devices). Antioxidant activity was calculated from the vitamin C standard curve and expressed as vitamin C equivalents.

### 2.8 GC–MS Analysis

GC–MS analysis was performed using an Agilent 6890 Plus gas chromatograph coupled to a 5977 N quadrupole mass selective detector (Agilent Technologies; Palo Alto, CA, USA). Compounds were separated on a J&W Scientific capillary column (30 m × 0.25 mm i.d., 0.25 μm film thickness) coated with a 5% diphenyl/95% dimethylsiloxane stationary phase. The instrument was operated in electron impact ionization mode at 79 eV. The injection port and MS interface temperatures were maintained under controlled conditions, and mass spectra were acquired in full‐scan mode over an m/z range of 50–700.

### 2.9 In Vivo Experiment I and II: Intervention Studies Using *S. crispa* Extract and Its Active Compound, Ergosterol

Five‐week‐old male ICR (Institute of Cancer Research) mice were obtained from Daehan Biolink Co. (Eumseong, Republic of Korea) and acclimated to the animal facility for five days prior to intervention. Randomization was performed using a computer‐generated schedule to minimize allocation bias. The mice were housed four per cage under controlled environmental conditions (23°C ± 1°C, 55% relative humidity, 12 h light/dark cycle) with free access to standard chow and water. All mice were maintained on a standard commercial diet (Daehan Biolink), which contained crude protein ≥ 20.5%, crude fat ≥ 3.5%, crude fiber ≤ 8.0%, crude ash ≤ 8.0%, calcium ≥ 0.5%, and phosphorus ≥ 0.5%. For crude extract treatment (In vivo experiment I), *S. crispa* extract was incorporated into the standard chow diet at doses of 400, 800, and 1200 mg/kg body weight. The extract dose levels (400, 800, and 1200 mg/kg body weight) were selected based on previously reported in vivo studies of edible and medicinal mushroom extracts and polysaccharide‐rich preparations, which commonly employ oral dose ranges of approximately 400–1000 mg/kg to evaluate biological efficacy while maintaining tolerability (Friedman 2016; Wasser 2011). For example, mushroom‐derived polysaccharides have been administered at 400 and 800 mg/kg in murine models with documented systemic effects and acceptable safety profiles (Friedman 2016). Broader reviews of medicinal mushroom research likewise indicate that several hundred mg/kg represents a frequently adopted efficacious range for crude mushroom‐derived preparations in rodents (Wasser 2011). Accordingly, 400 mg/kg was selected as a lower biologically relevant dose within this commonly used range, 800 mg/kg as an intermediate dose, and 1200 mg/kg as a higher dose to assess potential dose–response effects beyond the typical 400–800 mg/kg window while remaining within a generally tolerable range reported for mushroom polysaccharides. Under our experimental conditions, ergosterol supplementation did not induce detectable hepatic toxicity, as indicated by unchanged serum ALT and AST levels. For the *S. crispa* extract groups, no overt signs of systemic toxicity (e.g., abnormal behavior or body weight loss) were observed during the study period. For ergosterol intervention, a putative bioactive compound of *S. crispa* extract (In vivo experiment II), the compound was thoroughly mixed with the chow diet at 10, 20, and 40 mg/kg body weight. For both experiments, to induce neurotoxicity, a single intraperitoneal (i.p.) injection of trimethyltin chloride (TMT) was administered, and behavioral assessments were conducted thereafter to evaluate the cognitive‐enhancing effects of *S. crispa* and ergosterol. The Y‐maze test was performed 2 days after TMT injection, followed by the passive avoidance test 3 days later. For euthanasia, mice were humanely sacrificed by terminal cardiac puncture performed under deep avertin anesthesia, ensuring that the procedure was conducted without pain or distress. All animal procedures adhered to the guidelines of the Institutional Animal Care and Use Committee of Korea University (KUIACUC‐2017‐142).

### 2.10 Y‐Maze Test

The Y‐maze test was performed 3 days after TMT injection to evaluate short‐term spatial working memory. The apparatus, constructed from black acrylic, consisted of three identical arms (33 cm in length, 10 cm in width, and 15 cm in height) positioned at 120° angles. Each mouse was gently placed at the end of one arm and allowed to explore all three arms for 8 min freely. An arm entry was counted when all four limbs of the mouse were completely inside an arm. Spontaneous alternation behavior was defined as successive entries into three different arms in overlapping triplet sequences. The percentage of spontaneous alternation was calculated using the formula: [Number of actual alternations/(Total arm entries −2)] × 100.

### 2.11 Passive Avoidance Test

The passive avoidance test was performed to assess aversive learning and memory. The apparatus consisted of two adjoining chambers—one illuminated and one dark—both equipped with a stainless‐steel grid floor capable of delivering a mild foot shock. During the acquisition trial, each mouse was placed in the illuminated compartment, and upon entering the dark chamber, it received a brief electrical foot shock (0.5 mA for 1 s). Twenty‐four hours later, a retention trial was conducted by reintroducing the mouse into the illuminated compartment. The latency to re‐enter the dark chamber (step‐through latency) was recorded as a measure of memory retention, with a maximum cutoff time of 300 s.

### 2.12 Biochemical Assays

After completion of all behavioral tests, the mice were sacrificed, and blood samples were collected. Serum was separated by centrifugation at 4000 rpm for 15 min and stored at −80°C until analysis. Acute liver toxicity was assessed by measuring serum alanine aminotransferase (ALT) and aspartate aminotransferase (AST) levels using a commercially available transaminase reagent kit (ASAN Pharmaceutical; Seoul, Republic of Korea), following the manufacturer's instructions. Brains were rapidly removed, homogenized in ice‐cold buffer, and stored at −80°C for subsequent biochemical assays. AChE activity in brain homogenates was determined as previously described. Lipid peroxidation was evaluated using a thiobarbituric acid–based assay, in which 480 μL of 1% (v/v) phosphoric acid was added to the homogenate, followed by 160 μL of 0.67% (w/v) thiobarbituric acid solution. The mixture was heated at 95°C for 45 min and then extracted with *n*‐butanol. The absorbance of the organic phase was measured at 532 nm using a microplate reader (SpectraMax M, Molecular Devices). Lipid peroxidation levels were expressed as malondialdehyde (MDA) equivalents.

### 2.13 Statistical Analysis

All data are presented as the mean ± standard deviation (SD). Normality was assessed using the Shapiro–Wilk test. For data that did not meet the assumption of normality, the Kruskal–Wallis test followed by Dunn's multiple comparison test was applied. Statistical significance for normally distributed data was evaluated using one‐way analysis of variance, followed by Dunnett's post hoc test (vs. TMT group). A *p* value < 0.05 was considered statistically significant. All statistical analyses were performed using GraphPad Prism version 8.0.2 (GraphPad Software, San Diego, CA, USA).

## 3 Results and Discussion

### 3.1 Screening of Plant Extracts for AChE Inhibitory Activity

To identify natural sources with potential cholinesterase‐modulating activity, ethanolic extracts from various plant materials were initially screened for AChE inhibitory activity at a concentration of 1 mg/mL (Table 1). Tacrine (100 nM) was used as a positive control to validate assay performance. While several extracts showed marginal or moderate inhibition, the AChE inhibitory effect of *S. crispa* was robust across independent experiments, indicating a reproducible bioactivity. Importantly, the observed activity was detected at the crude extract level, implying the presence of one or more bioactive compounds capable of modulating cholinergic function. Based on its superior inhibitory profile in the primary screening, *S. crispa* was subjected to further anti‐oxidative capacity assays as well as GC–MS to identify bioactives therein.

### 3.2 *S. crispa* Extract Inhibits AChE Activity and Protects PC12 Cells From Oxidative Stress, and Ergosterol Is Identified as a Major Active Constituent

**FIGURE 1:**
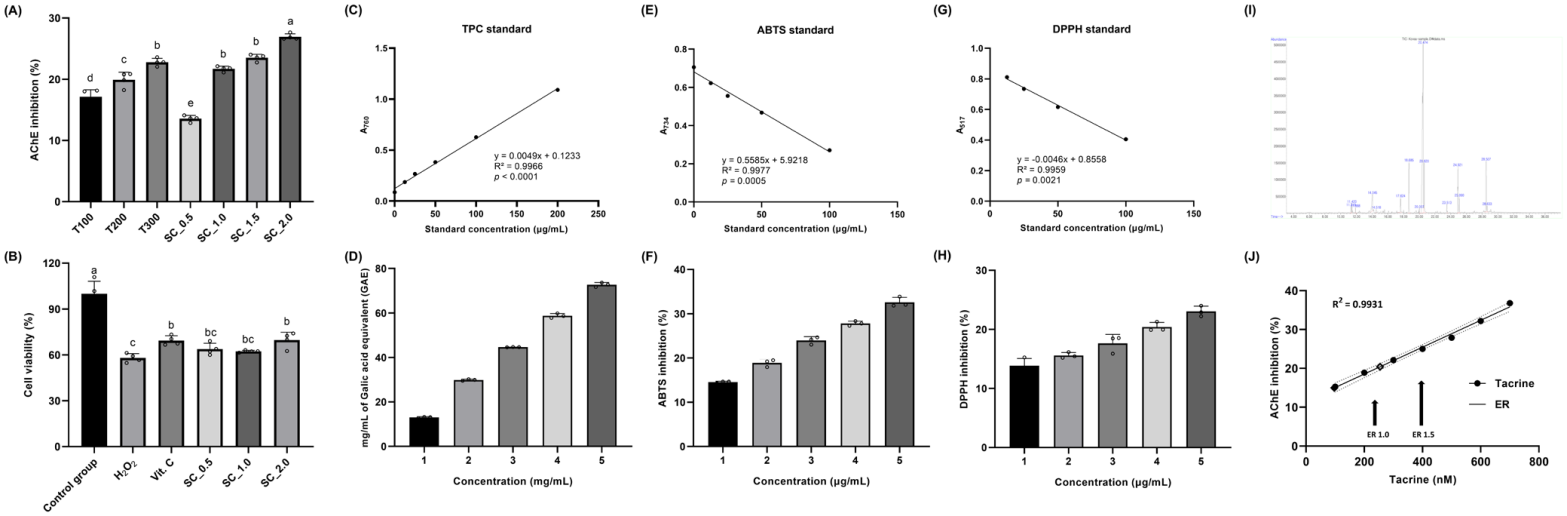
Bioactive properties of *Sparassis crispa* (SC) extract and identification of major constituent therein. (A) Acetylcholinesterase (AChE) inhibitory activity of the SC extract. Tacrine was used as a positive control at 100, 200, and 300 nM (T100, T200, and T300), while the SC extract was tested at 0.5, 1.0, 1.5, and 2.0 mg/mL (SC_0.5, SC_1.0, SC_1.5, and SC_2.0, respectively); (B) Protective effects of the SC extract against H_2_O_2_‐induced cytotoxicity in rat pheochromocytoma cells. Cells were pretreated with the SC extract (0.5, 1.0, and 2.0 mg/mL) for 48 h prior to exposure to 200 μM H_2_O_2_. Vitamin C (100 μM) was used as a positive control; (C) Gallic acid standard curve used for the determination of total phenolic content (TPC); (D) TPC of the SC extract expressed as mg gallic acid equivalents (GAE); (E) Vitamin C standard curve for the 2,2′‐azino‐bis (3‐ethylbenzothiazoline‐6‐sulfonic acid) diammonium salt (ABTS) radical scavenging assay; (F) ABTS radical scavenging activity of the SC extract expressed as percentage inhibition; (G) Vitamin C standard curve for the 2,2‐Diphenyl‐1‐picrylhydrazyl (DPPH) radical scavenging assay; (H) DPPH radical scavenging activity of the SC extract expressed as percentage inhibition; (I) Representative gas chromatography–mass spectrometry (GC–MS) chromatogram of the SC extract, identifying ergosterol (ER) as a major bioactive constituent; (J) Tacrine dose–response curve (0–700 nM; *R* ^2^ = 0.9931) was used to estimate tacrine‐equivalent AChE inhibitory activity of ER where ER was tested at 1.0 and 1.5 mg/mL, and its inhibitory activity was interpolated onto the tacrine standard curve. Normality was assessed using the Shapiro–Wilk test. Statistical significance for panels (A, B) was evaluated using one‐way analysis of variance, followed by Dunnett's post hoc test. Data are presented as mean ± standard deviation (A, B: *N* = 4; D, F, H: *N* = 3). Different superscript letters indicate statistically significant differences among groups (*p* < 0.05). ABTS, 2,2′‐azino‐bis (3‐ethylbenzothiazoline‐6‐sulfonic acid) diammonium salt; AChE, acetylcholinesterase; DPPH, 2,2‐Diphenyl‐1‐picrylhydrazyl; ER, ergosterol; GAE, gallic acid equivalents; SC, *Sparassis crispa*; T100–300, tacrine (100–300 nM); TPC, total phenolic content; Vit. C, vitamin C.

As shown in Figure 1A, the ethanolic extract of *S. crispa* exhibited a dose‐dependent inhibitory effect on AChE activity when assessed using PC12 cells. *S. crispa* extract significantly inhibited AChE activity at concentrations ranging from 0.5 to 2.0 mg/mL, with higher concentrations producing progressively greater inhibition (*p* < 0.05). Tacrine, used as a positive control at 100, 200, and 300 nM, showed the expected concentration‐dependent inhibition, confirming the validity of the assay system. Notably, the AChE inhibitory activity of *S. crispa* extract at higher concentrations approached the inhibitory range observed for tacrine, indicating that SC contains component(s) capable of modulating cholinergic enzyme activity.

To evaluate whether the AChE inhibitory activity of *S. crispa* extract was accompanied by cellular protective effects, its ability to attenuate oxidative stress–induced cytotoxicity was examined in PC12 cells. As shown in Figure 1B, exposure to hydrogen peroxide (H_2_O_2_, 200 μM) markedly reduced cell viability compared with the untreated control group (*p* < 0.05). *S. crispa* extract (0.5–2.0 mg/mL) ameliorated H_2_O_2_‐induced cytotoxicity as assessed by the MTT assay where *S. crispa* extract (at 2.0 mg/mL) showed comparable protection compared to that of vitamin C (100 μM), a well‐established antioxidant control.

To characterize the antioxidant properties of *S. crispa* extract, TPC and radical scavenging activities (i.e., ABTS, and DPPH) were assessed. The gallic acid calibration curve for the TPC assay showed excellent linearity (*R* ^2^ = 0.9966, *p* < 0.0001), and *S. crispa* extract exhibited a concentration‐dependent increase in TPC, expressed as GAE (Figure 1C,D). In addition, *S. crispa* extract demonstrated significant free‐radical scavenging activity in both ABTS and DPPH assays. Using well‐fitted vitamin C standard curves (ABTS: *R* ^2^ = 0.9977, *p* = 0.0005; DPPH: *R* ^2^ = 0.9959, *p* = 0.0021), radical scavenging activity increased progressively with increasing extract concentrations (Figure 1E–H). These results indicate that *S. crispa* extract possesses substantial antioxidant capacity, which may contribute to its protective effects against oxidative stress–induced neuronal damage and its AChE inhibitory activity.

To identify the bioactive constituent(s) responsible for the observed AChE inhibitory and cytoprotective effects, the *S. crispa* extract was subjected to GC–MS analysis. As shown in Figure 1I, ergosterol was detected as a major peak in the chromatogram, indicating that it is a predominant component of the *S. crispa* extract. Based on its abundance and known biological relevance, ergosterol was selected for further evaluation as a candidate active compound contributing to the bioactivity of *S. crispa* . The AChE inhibitory activity of ergosterol was subsequently quantified by comparison with tacrine using a dose–response calibration curve. As shown in Figure 1J, tacrine exhibited a strong linear dose–response relationship (*R* ^2^ = 0.9931) over the tested concentration range (0–700 nM). When ergosterol was tested at concentrations of 1.0 and 1.5 mg/mL, its AChE inhibitory activity could be interpolated onto the tacrine standard curve, demonstrating that ergosterol effectively suppressed AChE activity in a concentration‐dependent manner. These results indicate that ergosterol is a functionally active AChE inhibitor and likely represents a key contributor to the cholinesterase‐modulating and neuroprotective effects observed for the *S. crispa* extract.

Ergosterol, a major fungal sterol, has been reported to exert neuroprotective effects relevant to AD. Previous studies demonstrated that ergosterol promotes neurite outgrowth and suppresses amyloid‐β production through ERK/CREB‐related signaling, and ameliorates AD‐like phenotypes in cellular and *Caenorhabditis elegans* models (Sillapachaiyaporn et al. 2024). In addition, ergosterol attenuates neuroinflammation and oxidative stress by suppressing pro‐inflammatory signaling pathways and enhancing neuronal survival mechanisms. In the present study, ergosterol isolated from *S. crispa* directly inhibited AChE activity, providing new evidence that ergosterol may also modulate cholinergic dysfunction. Taken together, these mechanistic observations prompted us to further evaluate whether ergosterol and *S. crispa* extract could translate into functional cognitive benefits in vivo, which were subsequently assessed using behavioral tests in a TMT‐induced mouse model.

### 3.3 *S. crispa* Extract and Ergosterol Ameliorate TMT‐Induced Behavioral Deficits In Vivo

No significant differences in body weight or brain weight were observed among the experimental groups in either experiment (Table 2), indicating that *S. crispa* extract and ergosterol administration did not produce overt systemic or developmental effects under the present conditions.

**TABLE 2:** Body and brain weights of ICR mice treated with *Sparassis crispa* (SC) extract and ergosterol (ER).

Groups	Body weight (g)	Brain weight (g)
Experiment I: *Sparassis crispa* (SC) extract		
Control group	34.43 ± 1.61	0.41 ± 0.06
Trimethyltin chloride (TMT) group	34.27 ± 2.02	0.34 ± 0.03
SC_400	35.08 ± 1.37	0.40 ± 0.06
SC_800	34.03 ± 2.00	0.36 ± 0.02
SC_1200	33.35 ± 1.88	0.35 ± 0.02
Experiment II: Ergosterol (ER)		
Control group	35.52 ± 1.84	0.49 ± 0.05
TMT	35.85 ± 1.86	0.50 ± 0.03
ER_10	34.61 ± 1.27	0.50 ± 0.02
ER_20	34.74 ± 1.73	0 49 ± 0.03

*Note:* Body and brain weights were measured at the end of the experimental period in two independent studies: Experiment I (SC extract) and Experiment II (ER). Data are expressed as mean ± standard deviation (*n* = 7–8 per group). Statistical analyses were performed separately for each experiment using one‐way analysis of variance, followed by Dunnett's post hoc test (vs. TMT group). No significant differences were observed among the groups within each experiment (*p* > 0.05 for all).

Abbreviations: ER, ergosterol; SC, *Sparassis crispa*; TMT, trimethyltin chloride.

**FIGURE 2:**
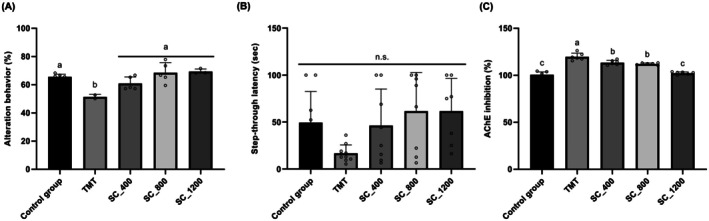
*Sparassis crispa* (SC) extract ameliorates trimethyltin chloride (TMT)‐induced cognitive impairment and restores cholinergic function in mice. (A) Y‐maze spontaneous alternation behavior, reflecting working memory performance; (B) Passive avoidance latency, evaluating aversive learning and memory retention; (C) Acetylcholinesterase (AChE) activity in mouse brain tissue following TMT exposure. Control mice received saline, whereas the TMT group received TMT (2.5 mg/kg, intraperitoneal injection). SC‐supplemented groups (400, 800, and 1200 mg/kg/day) were fed SC extract for 3 weeks prior to TMT injection. Normality was validated using the Shapiro–Wilk test. Statistical analyses were performed using one‐way analysis of variance, followed by Dunnett's post hoc test (vs. TMT group). Data represent mean ± standard deviation (*n* = 7–8). Different superscript letters indicate significant differences among groups (*p* < 0.05). AChE, acetylcholinesterase; n.s., not significant; SC, *Sparassis crispa*; TMT, trimethyltin chloride.

To determine whether the *S. crispa* extract translates its in vitro cholinesterase‐modulating and antioxidant activities into functional cognitive benefits in vivo, behavioral assessments were conducted using a TMT–induced mouse model. This model is widely employed to recapitulate key pathological features relevant to AD, including cholinergic dysfunction, oxidative stress, and cognitive impairment (Faryadras et al. 2025). Spatial working memory and learning performance were evaluated using the Y‐maze and passive avoidance tests, respectively as previously demonstrated (Kim et al. 2010). In the Y‐maze test, TMT administration significantly reduced spontaneous alternation behavior compared with the control group, indicating impaired working memory (Figure 2A). Dietary supplementation with *S. crispa* extract significantly improved alternation behavior, with mice receiving 800 or 1200 mg/kg exhibiting values comparable to those of the control group (*p* < 0.05 vs. TMT). These findings suggest that *S. crispa* extract effectively counteracts TMT‐induced working memory deficits, consistent with the cholinergic hypothesis that links cognitive impairment to disrupted acetylcholine signaling. In contrast, although TMT‐treated mice displayed a marked reduction in step‐through latency in the passive avoidance test—reflecting deficits in learning and memory retention (Figure 2B)— *S. crispa* supplementation only showed a modest tendency toward latency prolongation, which did not reach statistical significance under the present experimental conditions. This differential outcome between behavioral paradigms suggests that the cognitive benefits of *S. crispa* extract may be more pronounced in tasks assessing spatial working memory than in aversive learning, potentially reflecting task‐specific sensitivity to cholinergic modulation. Consistent with the behavioral outcomes, TMT exposure significantly elevated AChE activity in mouse brain tissues compared with controls (Figure 2C). *S. crispa* extract supplementation significantly attenuated this TMT‐induced increase in AChE activity, particularly in mice receiving 400 or 800 mg/kg (*p* < 0.05 vs. TMT). These results support the notion that restoration of cholinergic homeostasis contributes, at least in part, to the observed improvements in cognitive performance, and align with the established role of AChE inhibition in symptomatic management of cognitive decline (Colovic et al. 2013).

**FIGURE 3:**
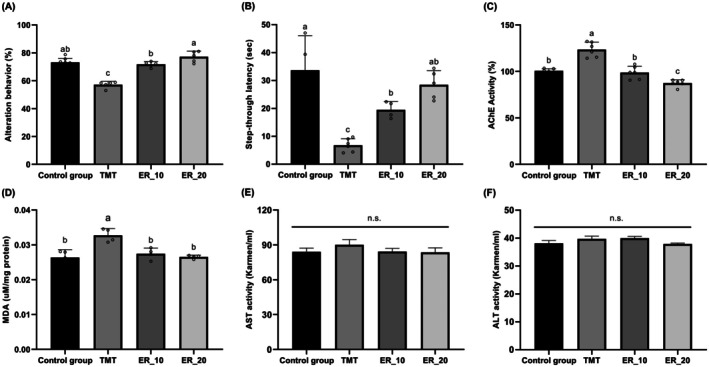
Ergosterol (ER) ameliorates trimethyltin chloride (TMT)‐induced cognitive deficits, oxidative stress, and cholinergic dysfunction in mice. Effects of ER supplementation on (A) Y‐maze alternation behavior, (B) passive avoidance test, (C) acetylcholinesterase (AChE) activity in mice brain tissues, (D) lipid peroxidation (malondialdehyde, MDA) levels in mice brain tissues, and (E, F) serum aspartate aminotransferase (AST) and alanine aminotransferase (ALT) activities. The control group received saline injections, whereas the TMT group received TMT (2.5 mg/kg body weight, intraperitoneal injection). ER‐supplemented groups (ER_10 and ER_20) were administered TMT after 3 weeks of dietary ER supplementation (10 or 20 mg/kg/day, respectively). Normality was assessed using the Shapiro–Wilk test, and significance was evaluated using one‐way analysis of variance, followed by Dunnett's *post hoc* test. Results are shown as means ± standard deviations (*n* = 7–8). Different superscripts indicate significant differences among groups at *p* < 0.05. AChE, acetylcholinesterase; ALT, alanine aminotransferase; AST, aspartate aminotransferase; ER, ergosterol; n.s., not significant; TMT, trimethyltin chloride.

Based on the identification of ergosterol as a major bioactive constituent of *S. crispa* extract and its demonstrated AChE inhibitory activity in vitro, the cognitive effects of ergosterol were further evaluated in the same TMT‐induced mouse model (Figure 3A,B). In the Y‐maze test, TMT‐treated mice again exhibited a significant reduction in spontaneous alternation behavior relative to control mice (Figure 3A). Ergosterol supplementation significantly improved alternation performance in a dose‐dependent manner, with mice receiving 20 mg/kg showing alternation levels comparable to those of the control group (*p* < 0.05 vs. TMT). This dose‐responsive effect reinforces the role of ergosterol as a key contributor to the cognitive benefits associated with *S. crispa* extract. Similarly, in the passive avoidance test, TMT administration resulted in a pronounced decrease in step‐through latency, indicating impaired learning and memory retention (Figure 3B). Ergosterol supplementation significantly prolonged latency times, with the higher dose (20 mg/kg) exerting a more pronounced protective effect than the lower dose (10 mg/kg). Unlike the crude extract, ergosterol produced significant improvements in both behavioral paradigms, suggesting that isolation of the active component enhances efficacy in tasks requiring sustained memory retention.

To further explore the biochemical basis underlying these behavioral improvements, markers of cholinergic function and oxidative stress were assessed in mouse brain tissues. TMT administration significantly increased AChE activity compared with the control group (Figure 3C), whereas ergosterol supplementation significantly suppressed AChE activity, restoring levels toward those observed in control mice (*p* < 0.05 vs. TMT). In parallel, lipid peroxidation—assessed by MDA levels—was significantly elevated in the brains of TMT‐treated mice (Figure 3D). Ergosterol supplementation significantly reduced MDA levels in mice receiving 10 or 20 mg/kg, indicating attenuation of TMT‐induced oxidative damage. These findings highlight the dual action of ergosterol on both cholinergic dysfunction and oxidative stress, two interrelated pathological features implicated in AD progression (Francis et al. 1999).

Finally, to address safety considerations critical for translational application, potential systemic toxicity of ergosterol was evaluated by measuring serum AST and ALT activities (Figure 3E,F). No significant differences in AST or ALT levels were observed among the experimental groups, indicating that ergosterol supplementation did not induce hepatic toxicity under the present experimental conditions (Ozer et al. 2008). This safety profile further supports the potential of ergosterol and *S. crispa* extract more broadly as candidates for development as functional food‐based interventions preventing cognitive decline.

## 4 Conclusion

In the present study, we demonstrated that *S. crispa* extract exerts AChE inhibitory and antioxidant activities in vitro, and that these bioactivities translate into functional cognitive benefits in vivo. We further identified ergosterol as a major bioactive constituent of *S. crispa* extract that is responsible for cholinesterase modulation and neuroprotective effects. These findings provide mechanistic insight into the previously reported cognitive benefits of *S. crispa* (Zhang et al. 2023) and address a critical knowledge gap regarding its active components. Compared with previous studies reporting general antioxidant or neuroprotective properties of *S. crispa* , the present study identifies ergosterol as a major bioactive constituent associated with acetylcholinesterase inhibition and cognitive improvement. Although detailed molecular mechanisms were not investigated, the combined in vitro and in vivo findings suggest a functional linkage between sterol components and cholinergic modulation. By integrating phytochemical characterization with behavioral evaluation, this study narrows the gap between compositional analysis and functional outcomes. These findings extend the current understanding of *S. crispa* and provide a plausible biochemical basis for its potential role as a functional food–derived candidate for supporting cognitive health. Behavioral analyses using a TMT–induced mouse model revealed that dietary supplementation with *S. crispa* extract significantly improved spatial working memory and attenuated TMT‐induced cholinergic dysfunction. In parallel, ergosterol supplementation ameliorated cognitive deficits while reducing oxidative stress and restoring brain AChE activity, supporting its role as a key contributor to the in vivo efficacy of the crude extract. From a broader perspective, the dual modulation of cholinergic dysfunction and oxidative stress by *S. crispa* extract and ergosterol aligns with the multifactorial pathophysiology of AD (DeTure and Dickson 2019) and supports the concept of multi‐target natural products as complementary strategies for cognitive health. Furthermore, the absence of detectable hepatic toxicity in vivo underscores the safety profile of ergosterol under the experimental conditions, an important consideration given the adverse effects associated with current synthetic AChE inhibitors. Taken together, our findings position *S. crispa* and its bioactive compound ergosterol as promising functional food–derived candidates for the prevention or mitigation of cognitive decline. Future studies investigating long‐term efficacy, additional molecular mechanisms, and interactions among multiple bioactive constituents will further clarify their translational potential in the context of neurodegenerative disease prevention.

## Author Contributions

**Chan Kyu Park:** conceptualization, methodology, formal analysis, investigation, data curation, and writing – original draft preparation. **Gyumin Kang:** conceptualization, methodology, investigation, and data curation. **Soo Jung Choi:** methodology, formal analysis, and investigation. **Yoon Seo Lee:** methodology, formal analysis, and investigation. **Eui‐Cheol Shin:** methodology, data curation, formal analysis, and investigation. **Hyo In Kim:** methodology, data curation, and investigation. **Jinbong Park:** conceptualization, formal analysis, and investigation. **Nam Su Oh:** conceptualization, formal analysis, and investigation. **Hyung Taek Cho:** conceptualization, methodology, formal analysis, and investigation. **Tae Gyun Kim:** conceptualization, methodology, formal analysis, and investigation. **Jeong Hoon Pan:** conceptualization, formal analysis, and validation. **Dong‐Hoon Shin:** conceptualization, methodology, formal analysis, and investigation. **Young‐Jun Kim:** conceptualization, methodology, project administration, and supervision. **Jae Kyeom Kim:** conceptualization, methodology, formal analysis, funding acquisition, project administration, supervision and writing – review and editing.

## Funding

This research was supported by the Basic Science Research Program through the National Research Foundation of Korea funded by the Ministry of Education (RS‐2023‐00245564), by a grant from the Ministry of Food and Drug Safety (RS‐2024‐00332492), and by the Industry‐University‐Research Collaboration R&D program (Project No. 02311730) funded by the Ministry of SMEs and Startups (MSS), Republic of Korea. This study was supported by Korea University Grant (2025, Spring Semester).

## Ethics Statement

All animal experiments were conducted in accordance with the Guide for the Care and Use of Laboratory Animals and the ARRIVE guidelines. The protocol was approved by the Institutional Animal Care and Use Committee of Korea University (KUIACUC‐2017‐142).

## Conflicts of Interest

The authors declare no conflicts of interest.

## Data Availability

The datasets generated and/or analyzed during the study are available from the corresponding author on reasonable request.
